# A qualitative study of older adults' responses to sitting-time questions: do we get the information we want?

**DOI:** 10.1186/1471-2458-11-458

**Published:** 2011-06-10

**Authors:** Jannique GZ van Uffelen, Kristiann C Heesch, Robert L Hill, Wendy J Brown

**Affiliations:** 1 The University of Queensland, School of Human Movement Studies, Brisbane, Queensland 4072, Australia; 2 Queensland University of Technology, Institute of Health & Biomedical, Innovation and the School of Public Health, Brisbane, Queensland 4059, Australia

## Abstract

**Background:**

In the last decade, there has been increasing interest in the health effects of sedentary behavior, which is often assessed using self-report sitting-time questions. The aim of this qualitative study was to document older adults' understanding of sitting-time questions from the International Physical Activity (PA) Questionnaire (IPAQ) and the PA Scale for the Elderly (PASE).

**Methods:**

Australian community-dwelling adults aged 65+ years answered the IPAQ and PASE sitting questions in face-to-face semi-structured interviews. IPAQ uses one open-ended question to assess sitting on a weekday in the last 7 days 'at work, at home, while doing coursework and during leisure time'; PASE uses a three-part closed question about daily leisure-time sitting in the last 7 days. Participants expressed their thoughts out loud while answering each question. They were then probed about their responses. Interviews were recorded, transcribed and coded into themes.

**Results:**

Mean age of the 28 male and 27 female participants was 73 years (range 65-89). The most frequently reported activity was watching TV. For both questionnaires, many participants had difficulties understanding what activities to report. Some had difficulty understanding what activities should be classified as 'leisure-time sitting'. Some assumed they were being asked to only report activities provided as examples. Most reported activities they normally do, rather than those performed on a day in the previous week. Participants used a variety of strategies to select 'a day' for which they reported their sitting activities and to calculate sitting time on that day. Therefore, many different ways of estimating sitting time were used. Participants had particular difficulty reporting their daily sitting-time when their schedules were not consistent across days. Some participants declared the IPAQ sitting question too difficult to answer.

**Conclusion:**

The accuracy of older adults' self-reported sitting time is questionable given the challenges they have in answering sitting-time questions. Their responses to sitting-time questions may be more accurate if our recommendations for clarifying the sitting domains, providing examples relevant to older adults and suggesting strategies for formulating responses are incorporated. Future quantitative studies should include objective criterion measures to assess validity and reliability of these questions.

## Background

In the last decade, there has been increased interest in the health effects of sedentary behavior. The term 'sedentary behavior' refers to activities with low energy expenditure, typically ≤1.5 metabolic equivalents, such as sitting to watch television or to read [1,2]. There is a rapidly growing body of evidence suggesting that more time spent in sedentary behaviors is associated with increased health risks and poorer health outcomes, such as overweight and obesity, diabetes, and mortality, regardless of the time spent in physical activity (PA) [2-4].

Researchers have, therefore, suggested that epidemiological studies should include measures of both sedentary behavior and PA, in order to examine the associations between sedentary behavior, PA, and health [1]. In addition, surveillance of sedentary behaviors is important for monitoring trends over time [5]. In epidemiological studies and population surveillance, sedentary behavior is often conceptualized as 'sitting time' and self-report questionnaires are widely used to assess sitting time in these large studies [6].

It may be of particular interest to assess sitting time in older adults, as the proportion of older adults doing little or no PA increases with age [7,8] despite the health benefits of regular PA participation [9]. US surveillance data show that the highest proportion of community-dwelling adults reporting no PA, 30% in men and 40% in women, is found in those aged 75+ years [8] and that adults aged 70-85 years spend 9.3 hours per day in sedentary behaviors, more than any other adult age group [10]. Because replacing 30 minutes of sedentary time with light PA is associated with better physical health in adults aged 65+ years [11], focusing on reducing sitting time could be an effective strategy for improving health in older adults.

Although sitting-time questions are included in some PA surveillance questionnaires, little is known about older adults' understanding of these questions and the optimal format for sitting-time questions in this population. The aim of this study was therefore to document older adults' understanding of sitting-time questions using cognitive interviewing. Cognitive interviewing is a recognized method for identifying problems with survey questions, which usually do not become apparent in quantitative studies, and is often used to evaluate how well questions are meeting their objectives [12,13]. Two approaches to asking about sitting time were assessed in this study, in order to evoke different responses: 1) an open-ended, single question asking for sitting 'at work, at home, while doing coursework and during leisure time', as used in the International PA Questionnaire (IPAQ); and 2) a closed three-part question asking for leisure-time activities only, as used in the PA Scale for the Elderly (PASE).

## Methods

### Participants

Previous research using cognitive interviewing methods has indicated that the number of problems identified in a questionnaire is associated with the sample size, with the highest number of uncovered problems for a sample size of 50 [14]. To collect useful cognitive interview data from at least 50 participants, we recruited 55 community-dwelling adults, aged ≥65 years, from Brisbane, Australia. Eligibility criteria included the ability to walk > 100 meters without aid, to speak and understand English, and to be cognitively able to respond to our questions. Participants were purposefully selected to ensure representation of men and women of different age groups, PA levels, and education levels, given the influence of these factors on questionnaire comprehension [15]. Further recruitment details have been reported elsewhere [16]. The study protocol was approved by the University of Queensland Medical Research Ethics Committee.

### Data collection protocol

Participants were mailed a questionnaire asking for socio-demographic and health-related characteristics and an informed consent form, both of which they completed and submitted at the start of a face-to-face interview. Each interview was conducted by two researchers (JvU and KH, or KH and RH): one asked the study questions, while the other was responsible for recording the interviews, noting participants' non-verbal communication and conducting probing when needed. JvU and KH were trained in survey methodology and interviewing techniques as part of their master's and doctorate degrees. RH, a masters student gaining experience in cognitive interviewing techniques during this project, first observed the interviews with JvU and KH and later took notes and did additional probing with KH interviewing participants.

During the interview, participants responded to questions from four short PA questionnaires, two (PASE and IPAQ) of which included sitting questions. A computer-based random order generator was used to assign the order in which the participants received the questionnaires. To decrease possible bias resulting from the questionnaire order, participants were instructed, before the start of each new questionnaire after the first one, to respond as if they had not already responded to similar questions. For the current analysis, cognitive interview data from all 55 interviewed participants were used. This study expands our previous analysis of IPAQ, which used a subsample of these 55 participants [16].

### Cognitive interviews

Conrad's question-and-answer model of survey response [17] was used as the theoretical framework for the cognitive interviews. Based on this model, an interview protocol was developed to follow participants' movement through three stages of responding to a question: 1) understanding the intent of the question (comprehension of what information is requested and what process should be used to retrieve that information); 2) response formulation (conducting the mental operations required to formulate a response, including information retrieval, mental arithmetic and evaluation of a response); and 3) response formatting (mapping of a response to pre-specified response options). Two cognitive interviewing techniques were used: 1) concurrent think-aloud, in which participants were asked to express their thoughts as they answered questions, followed by 2) probing, using scripted and unscripted follow-up questions [12,13,18]. The interview protocol was tested in three practice interviews and refinements to the protocol were made after each practice interview.

The interview began with a general introduction to the study and time for participants to practice using the 'think-aloud' technique. Participants were then asked to read out loud the questions and use this technique whilst formulating an answer to each sitting question before writing their answers on an answer sheet. In line with recommendations, the researcher who asked the questions encouraged the participants to verbalize their thoughts while answering the questions, but intervened as little as possible during the 'think aloud' process [12]. Any problems with using the response formats were noted by the other researcher. When participants did not provide sufficient details while thinking aloud, the interviewer probed for greater insight into participants' comprehension (*'What activities are you including in your answer?'*), for information about response formulation (*'How did you come up with this answer?'*), and for clarification. Participants were then asked to describe any activities they did not include in their answers and the reasons why (i.e., any sitting activities they performed but did not include in their responses). At the interview completion, participants received a $20 gift voucher.

### International Physical Activity Questionnaire (IPAQ)

The self-report short form of IPAQ was used [19]. The IPAQ sitting question asks about the 'time spent sitting on a week day, during the last 7 days', in the following domains: 'at work, at home, while doing coursework and during leisure time'. IPAQ has an open-ended response and participants are asked to report 'hours per day' and 'minutes per day'. Participants were given the option to check a box below the spaces for these responses to indicate 'don't know/not sure'. IPAQ was initially validated for use with adults aged 18-65 years from 12 countries [20], and the validity of the sitting item was examined with a subsample from that study [5]. Test-retest reliability ranged from 0.62-0.96 (Spearman's ρ) and validity against an accelerometer was 0.34 (pooled Spearman's ρ). In a South African study of adults aged 60+ years, 3- to 5-day test-rest reliability of sitting time was 0.76, and criterion validity against accelerometers was -0.32 for time and -0.45 for total counts (all Spearman's ρ) [21]. In a Swedish study examining a modified version of the IPAQ for people aged 65+ years, criterion validity of the IPAQ sitting item against accelerometers was 0.28 (Spearman's ρ) [22].

### Physical Activity Scale for the Elderly (PASE)

The PASE sitting question consists of three parts. The first part asks about frequency of sitting in 'leisure time activities': 'Over the past 7 days, how often did you participate in sitting activities, such as reading, watching TV or doing handcrafts'? The responses categories are 'never, seldom (1-2 days), sometimes (3-4 days) and often (5-7 days)'. The second part is open ended and asks for the specific sitting activities ('What were these activities?'). The final part of the question asks about the duration of those activities: 'On average, how many hours per day did you engage in these sitting activities?' The response categories for this question are 'less than 1 hour, 1 but less than 2 hours, 2-4 hours and more than 4 hours.'

PASE was developed, and its PA questions were initially validated, in the US and it is now used internationally for epidemiological studies of PA in adults aged 65+ years [23]. Items included in the PASE activity summary scales assess occupational, household and leisure-time PA in the previous 7 days. The original validation study indicated significant, although low to moderate, correlations between these PA summary scores and physiological measures and health status (correlations ranged from *r *= -0.13 for resting heart rate to *r *= -0.42 for a sickness impact profile score) [23]. Subsequently, PA summary scores have been validated against energy expenditure measured by doubly-labeled water (*r *= 0.58) [24], and against accelerometer PA counts (r = 0.49) [25]. Because sitting data are not included in the PA summary scores, these data have not been included in reliability and validity testing.

### Data analysis

Interviews were audio-recorded and professionally transcribed. NVivo 8 qualitative analysis software (QSR International, Melbourne, Australia) was used to organize, manage and code the data. Initially, each word processing file containing an interview transcript, along with the notes taken during the interview, was imported into NVivo. Prior to importing the data into NVivo, all the data within each file had been labeled to reflect the questionnaire to which they pertained as well as the specific activity they addressed (e.g., PASE sitting items; IPAQ sitting items). The importation process then automatically created codes in NVivo for each of the labels, to allow for the coding of data separately by activity within each questionnaire.

For these analyses, participants' responses to sitting questions were coded into the understanding, response formulation, and response mapping stages according to Conrad's model of survey response [17]. This model postulates that within each of these stages, computational, lexical, logical, omission, or temporal problems may be evident [17]. Therefore, we specifically looked for evidence of such problems within each stage and developed initial themes that reflected the problems we found within each stage. In addition, we created a separate 'Activities' theme to describe the sitting activities that were reported.

Three researchers (JvU, KH, RLH) jointly developed the initial themes within each stage, using sitting data from participants who completed the IPAQ or PASE as their first survey. Next, coding rotated among members of the research team with sitting data from each participant being coded by two members, using the initial themes. Discrepancies between coders were discussed in team meetings and consensus was used to determine the final coding. Finally, JvU reviewed all themes, merged those that overlapped, and summarized the findings, which were then reviewed by KH to ensure that the major themes were captured.

## Results

Characteristics of the sample are presented in Table 1. Women and men were well represented across age categories. Most participants were born in Australia and had university degrees. Although all participants had reached retirement age, a few were still employed. Most participants reported that it was easy or not too difficult to manage on their income, but a few reported this was difficult or impossible. The only demographic variable that differed significantly between men and women was marital status: a higher percentage of men than women were married (p < 0.05). All participants considered themselves healthy, but a few reported limitations in walking 500 meters.

**Table 1:** Characteristics of participants ^a^

	Men (n = 28)		Women (n = 27)		Total (N = 55)	
	
	n	(%)^b^	n	(%)^b^	n	(%)^b^
**Demographics**						
Age, years						
65-69	8	(29)	10	(37)	18	(33)
70-74	11	(39)	8	(30)	19	(35)
≥75	9	(32)	9	(33)	18	(33)
Country of birth						
Australia	19	(68)	15	(56	34	(62)
Other English-speaking country	8	(29)	9	(33)	17	(31)
Non-English-speaking country	1	(4)	3	(11)	4	(7)
Education						
No tertiary education	3	(11)	3	(11)	6	(11)
Certificate or trade	10	(36)	9	(33)	19	(35)
University degree or higher	15	(54)	15	(56)	30	(55)
Employment						
Employed	2	(7)	3	(11)	5	(9)
Retired/not employed	26	(94)	24	(89)	50	(91)
Income management						
Easy	13	(46)	10	(37)	23	(42)
Not too bad	13	(46)	11	(41)	24	(44)
Difficult/impossible	2	(7)	6	(22)	8	(15)
Marital status						
Married/common-law marriage	**22**	**(79)**	**14**	**(52)**	**36**	**(66)**
Widowed/never married/separated/divorced	**6**	**(21)**	**13**	**(48)**	**19**	**(35)**
**Health**						
Self-rated health						
Excellent	9	(32)	5	(19)	14	(26)
Very good	8	(29)	16	(59)	24	(44)
Good	11	(39)	6	(22)	17	(31)
Limited in walking 500 m						
Limited a little	2	(7)	3	(11)	5	(9)
Not limited	26	(93)	24	(89)	50	(91)

**^a ^**Boldface numbers indicate significant difference between men and women (chi-square, p < 0.05).

**^b^**Cells may not add up to 100% due to rounding.

### Sitting activities

For IPAQ, participants reported various sitting activities, as shown in Table 2. Almost all participants reported sitting to watch TV. Many men reported sitting in front of a computer, while few women did. Other commonly reported activities were mental activities, most frequently reading, but also playing bridge and doing crosswords. Many participants reported sitting to eat meals. A few men and women reported transport-related sitting. Some counted lying down for resting/napping as sitting, and a few women, but no men, reported sitting to do handcrafts.

**Table 2:** IPAQ and PASE sitting questions and activities reported by participants

	IPAQ		PASE	
***Questionnaire specifications***				

**- Behavior examined**	Sitting on a week day in last 7 days		Sitting per day in last 7 days	

**- Domain**	Sitting at work, at home, while doing course work and during leisure time		Leisure time activity	

**- Examples**	Sitting at a desk, visiting friends, reading, or sitting or lying down to watch television		Reading, watching TV, or doing handcrafts	

	**IPAQ**		**PASE**	

	**Women**	**Men**	**Women**	**Men**

***Activities reported, as asked about in examples***				

**- Sitting at a desk**		- working at desk		

**- Visiting friends**			- social activities	- social activities
	- visits		- visits	

**- Reading**	- reading	- reading	- reading	- reading

**- TV**	-TV	- TV	- TV	- TV

**- Crafts**	- knitting		- handcrafts	
			- cross stitching	
			- crocheting	
	- knitting		- knitting	
			- mending	
			- painting	
	- sewing		- sewing	
	- silversmithing		- silversmithing	

***Activities reported, not asked about in examples***				

**- Other mental **	- board games			
**activites**			- book club	
	- bridge	- bridge	- bridge	- bridge
	- computer	- computer	- computer	- computer
	- crossword	- crossword	- crossword	- crossword
		- meetings		
	- paperwork	- paperwork		- paperwork
			- piano	
		- puzzles	- puzzles	
	- studying	- studying	- studying	- studying
			- Sudoku	
	- volunteer work			
	- writing	- writing	- writing	

- **Transport-related**	- in car	- in car		- in car
**activities**	- in plane			
	- waiting at airport			

- **Other social and**	- eating/meals	- eating/meals	- eating/meals	- eating/meals
**relaxation activities**				- concert
	- going out		- going out	
	- listening to radio	- listening to radio		
	- resting/napping	- resting/napping		
	- sitting in sun			
	- theatre			
				- telephoning

IPAQ = International Physical Activity Questionnaire; PASE = Physical Activity Scale for the Elderly.

For PASE, most women reported combinations of watching TV, reading and other mental activities, and handcrafts (see Table 2). Men reported the same activities, except for handcrafts. As was the case for IPAQ, almost all participants reported sitting to watch TV, and the majority reported reading. Other mental activities, such as studying, were reported by many men, but by few women. Instead, women tended to report in detail about handcrafts. As was found for IPAQ, only a few participants reported sitting during transport for PASE. In contrast to IPAQ, only a few participants reported sitting for meals for PASE.

For both IPAQ and PASE, some participants reported doing multiple sitting activities at the same time. For example, one woman said for PASE, *"The knitting and the TV watching, yeah, they combine because I mean I just sit there and knit."*

### Comprehension of the requested information

#### Understanding the meaning of the words in the question

Only a few participants misunderstood the meaning of the words used in the sitting questions. For IPAQ, one man wondered about the use of the word 'weekday,' and concluded that the survey must have been developed for working people. For PASE, a few participants expressed confusion about the meaning of 'sitting activities,' and thought this meant they were being asked to include *"special cases of sitting," *not regular daily sitting activities such as eating. The heading 'leisure time activity' before the PASE sitting question also caused confusion for some participants. For example, one woman wondered, *"Is eating a leisure-time activity? Or no? I obviously did sit down to eat, and I don't know if that's a leisure-time activity?" *Another woman assumed that only sitting activities during daylight should be included, because PASE asks about hours *per day *spent in sitting activities.

#### Understanding the scope of sitting activities to report

As expected, participants used the domains described in the questions to guide their responses. Because PASE asks about leisure-time activities only, some participants did not report time spent in sitting activities they did not consider to be 'leisure'. In contrast, IPAQ instructs participants to include sitting 'at work, at home, while doing course work, and during leisure-time'. Therefore, some activities reported for IPAQ were not reported for PASE. For example, one woman said for PASE, *"Well, I'm not going to put reading in there because... I'm working when I'm reading." *For IPAQ, however, she reported her reading, and as a consequence, reported the most time spent sitting of all participants (13 hrs). Of particular note, sitting down for meals and for doing office work were more frequently reported for IPAQ than for PASE.

Not only the domains but also the examples given in the questions guided the responses. Many participants only reported time spent in sitting activities included as examples in the questions and not in other activities relevant to the question. This was more of a problem for PASE, for which many participants only reported sitting for reading, watching TV or doing handcrafts. One participant commented about her answer, *"It would have been TV, reading and handcrafts. It doesn't say about sitting and having a cup of coffee or having a glass of wine, does it?"*

Especially for PASE, several participants questioned whether activities that were mentioned as examples but that did not always involve sitting should be included. A woman said, *"I like to study in bed. Do you call that sitting down? Would that [question] include sleeping? If I lie down and read, it would be sitting." *Activities done while lying down, including sleeping, resting, and reading in bed, were more often reported for IPAQ than for PASE. A likely explanation is that IPAQ includes as an example lying down to watch TV, while PASE does not. Participants may have inferred from IPAQ's instructions that lying down to read or rest also counted towards time spent sitting.

After completing each question, participants were asked whether they had sat to do any activities in the last week that they had not included in their answer and if so, why they had not included these activities. A frequently mentioned reason for not including activities was that these were not included in the question. For example, for IPAQ, one man said, *"I haven't included sitting in the car, driving places, [as]...it doesn't mention that;...on an average day I probably spend 30 minutes riding in the car." *For PASE, a few participants said they had not considered other leisure-time sitting activities than the ones mentioned in the question. One man said, for example, *"Computer, book club, friends: so yes there are quite a few other activities that I haven't thought of...." *Other leisure-time activities that participants had done, but did not report, included social activities, such as talking on the phone, visiting with friends or family, and going to the theatre or to a football match. Reasons given for not reporting time spent in these activities were that the activity was not regularly done (e.g., going to the theatre), the activity also involved getting up and moving around (e.g., playing bridge), or the activity was not considered to be important for a participant (e.g., attending a meeting). Although only a few participants included time spent sitting during transport in their answer, the number of participants mentioning that they had not included the time spent sitting in transport when probed was also small.

#### Understanding the timeframe of sitting activities to report

Although both surveys ask about sitting activities in the last 7 days, most participants did not limit their responses to activities in this timeframe. Instead, using words like *"usually," "routine" *and *"habit," *they tended to report activities they did as part of daily life. One participant explained why he did not think about the previous week in particular by saying, *"Because I do it every day: every day I do watch the news and things like that."*

### Formulation of a response

#### Formulation of a response to sitting frequency

The frequency of sitting was assessed in PASE only. Participants easily responded to the PASE question about how often they sat down for leisure-time activities in the last 7 days. With sitting considered part of daily life, almost all participants selected 'often (5-7 days).' One woman said, *"I can't imagine that I consider sitting down sometimes [3-4 days] or never, people do sit often."*

#### Formulation of a response to sitting duration

Recalling time spent sitting was challenging for most participants. Many participants reported that, because sitting happened throughout the day, it was difficult to recall and sum. One participant said, *"It's fragmented time rather than a block of time in which you are doing an activity: it's much harder to add up." *Participants used a range of strategies to compute time spent sitting. While there was some overlap in strategies used for IPAQ and PASE, some strategies were questionnaire-specific, and a wider range of strategies was used for IPAQ than for PASE (Table 3).

**Table 3:** Strategies participants used for determining duration of sitting time

Strategy	IPAQ (open question)	PASE (closed question)
Thinking of minimum time usually spent sitting	-	**
Summing sitting time on a usual day	***	**
Determining the proportion of a usual day spent sitting	**	-
Subtracting all non-sitting activities in a usual day from 24 hours	*	-
Calculating average daily sitting time across a week	**	*
Picking the day for which sitting time is easiest to recall	*	-
Reporting sitting time for the 'most usual' day	*	*
Reporting sitting time for the day with highest time spent sitting	*	*
Using a combination of the above strategies	*	*
Reporting average/usual sitting time without doing calculations	**	*
Guessing time spent sitting	**	*
No strategy, question considered too difficult to answer	**	-

***: frequently mentioned strategy (majority participants); ** strategy mentioned by fewer participants (less than majority, but more than 5); * strategy mentioned by few participants (less than 5); - strategy not mentioned

A frequently used strategy for PASE was selecting the response option that included the minimum time usually spent sitting on a usual day, not specifically during the previous week as stated in the sitting-time questions. One woman who chose the option ' > 4 hrs/day' said, *"4 hours is very little...I would on the whole do 5 hours at night alone, and more often than not in the day time I'm sitting down." *This strategy was most often used by participants who reported sitting ' > 4 hrs/day', although a few participants used this strategy to select other response categories (i.e., 1-2 hrs or 2-4 hrs). Another common strategy for PASE was summing the time spent doing specific, habitual sitting activities on a usual day.

Summing the time spent doing specific sitting activities on a usual day was the most frequently used strategy for responding to the open-ended IPAQ question. For example, one woman said, *"I'm going to think about a typical day. So, I probably watched TV for about a couple of hours a day, and then I read for at least 2 hours, so that's 4. Then sitting down, having a meal or having a cup of tea or similar, that would be about 5." *Another common strategy for IPAQ was determining the proportion of a usual day spent sitting. For example, one man said, *"A day for me is probably approximately 14 hours and you spend half of that time on your feet and half of that time sitting." *A few people subtracted time in other activities from 24 hours, to arrive at their time spent sitting on a usual day. One man explained, *"Twenty-four hours in a day, I am sleeping from about 11 to about 5, so that is about 6 hours gone; 18 hours left of the day, I am doing about 3 of exercise, would you believe I spend 12 hours a day sitting down?*"

When schedules were not consistent across days, participants reported difficulties with calculating sitting time for one day. This was more of a problem for IPAQ because participants were asked to report time spent sitting on *a *week day, although some participants reported this problem for PASE as well. For example, after reading the PASE sitting question one woman said, *"This is where it's going to be a problem, if it's per day, because one day it was from 10 to 4, the other days it's less than an hour. So what do I do?" *She decided to sum her sitting time in the last 7 days and divide this by 7 to calculate an average daily sitting time. In contrast, most participants who calculated averages for PASE did so with a usual week in mind rather than the last 7 days. Likewise, for IPAQ only a few participants computed average sitting time in the last 7 days. Of these participants, only one reported average sitting time across the 5 weekdays (the questionnaire specifically asks about weekday sitting); the others averaged across 7 days. Other participants who reported sitting activities in the previous week solved the problem of differing daily sitting times using other strategies. These included picking a day for which it was easiest to recall sitting time, reporting sitting time for the 'most usual' day, or reporting sitting time for the day that they sat the most.

Participants used more complicated cognitive processes for determining sitting time for IPAQ than for PASE. This is illustrated by the need of some participants to use a combination of strategies for formulating a response to the IPAQ question, which was not evident for responding to the PASE questions. For example, one man reported:

"I'd sit to read the paper for an hour each day. I'd sit and read a book for 3 hours, so that makes 4 hours, and I sit and have a meal, so that's another, I suppose, 20 minutes, 3 times a day. So that's up to about 5 hours. Yeah, I would guess 5 hours, but I'm not terribly confident of that because I'm awake for 17 hours a day, and I'm not adding up to anything near 17. I mean, if I do it the other way around, 17 hours minus my vigorous and moderate activities I'd be sitting for 14 hours a day, but I can't remember sitting 14 hours a day. I can think of sitting for 5 hours. So I've got 14 hours to account for, so it's somewhere between 5 and 14 hours. I really find that a pretty difficult question to answer. I can only guess that it would be 7 hours a day, it might be 12."

Given the difficult cognitive processes required to respond to sitting questions, a number of participants guessed the time they spent sitting, particularly for IPAQ. For example, one woman said, *"...say 5 hours a day - 6? It's hard to say." *Several participants indicated that the IPAQ question was too difficult to answer. One man said, *"That's a very, very hard question. Look, I'm going to have to pull the pin on this one and say I don't know. I'd love to read some of the answers that some people give to that because that would be hilarious. I couldn't even work out a formula so I could come up with an answer on that one*."

### Mapping of a response to response options

Once participants had formulated their responses, few had difficulties mapping their response to pre-specified response options. However, because their daily sitting time was near the upper limit of one response option and the lower limit of another, two men circled two response options for the PASE question that asked about daily time spent sitting (one circled 1-2 hrs/day and 2-4 hrs/day, and the other circled 2-4 hrs/day and > 4 hrs/day). One woman asked if she could write down a range of daily sitting times for IPAQ.

## Discussion

Older adults typically spend much of their day sitting down [10]. Given the growing body of evidence on the ill-effects of too much sitting [3,4], it is important to include questions about sitting time in population surveillance of PA and epidemiological studies on sedentary behavior and health [1,5]. In this qualitative study, we document the cognitive processes used by a sample of older adults to respond to two widely used sitting-time questions. The results suggest that the ways that individual older adults answer sitting-time questions vary depending on (1) their understanding of the question, particularly the scope of the activities to be included and the timeframe to be considered; and (2) the strategies they employ to compute time spent sitting on a daily basis.

### Understanding the scope of sitting activities to report

Participants' perception of the scope of activities to include in their responses was largely guided by the examples provided. Many participants only reported activities indicated in the examples. This tendency was also observed in cognitive testing of another PA questionnaire in middle-aged US adults; some participants in that study considered the list of examples to be exclusive, rather than suggestive of activities to be included [26]. The complexity of the cognitive processing was decreased in that study by adding more varied examples to some questions and converting questions with many examples into multiple questions with fewer examples [26]. Although splitting questions could be a good strategy for obtaining more accurate information, short questionnaires are preferred for surveillance [20]. An option that may improve comprehensibility but would not affect the length of the questionnaire would be to add examples of sitting activities that are more relevant for older people, as was done in a Swedish study [22]. Additional qualitative research could determine appropriate activities to include.

Participants expressed difficulties conceptualizing 'sitting leisure activities'. This problem became more apparent for PASE than for IPAQ, probably because PASE exclusively asks about leisure-time sitting, whereas IPAQ also assesses other domains. Particularly for PASE, participants were unsure whether to include sitting while eating, resting/napping or driving, as these activities could be considered leisure-time activities, but were not included in the examples. Thus, some participants reported these activities while others did not.

Participants' perceptions of the scope of the activities to include also depended upon the domains from which the examples came. Neither questionnaire explicitly mentions transport or work as domains. Consequently, some participants reported, for example, transport-related sitting because they considered such sitting within the leisure-time domain, while others did not report this activity, as they did not consider it within the domains explicitly stated in the question. These findings suggest that respondents' understanding of the range of activities they are expected to report could be improved if they were given examples that reflect the full range of sitting activities in each domain. The results of this study further indicate that transport-related sitting is not an activity that people naturally consider in responding to questions about sitting activities. If respondents are expected to include transport-related sitting in their answers, they should be informed to do so. Clarifying domains and adding appropriate examples are likely to improve the accuracy of the responses, but further research is needed to evaluate the impact of doing so.

### Understanding the timeframe of sitting activities to report

Both IPAQ and PASE ask about sitting on a day in the last 7 days, with IPAQ specifying 'a weekday'. However, the majority of participants reported sitting for a usual day, or averaged sitting time across days in a usual week. Because many participants indicated that sitting time did not vary much across days or weeks, it was likely easier for them to recall their usual activities than to recall specific activities in the past 7 days that may not have been part of their usual week.

The issue of asking about a 'usual' or 'typical' week instead of the 'last 7 days' or 'last week' has been debated in physical activity epidemiology. For example, in the original 12-country validation study of IPAQ with predominately middle-aged adults, participants experienced difficulties with the interpretation of a usual week, and therefore, the use of the 'last 7 days' was recommended [20]. This is in line with earlier thinking in this field, which suggested that recall of actual PA participation during a set period (e.g. last 7 days) may provide more accurate estimates than recall of usual PA participation [27]. Our results suggest that this finding may not be applicable to recall of sitting time.

### Formulation of a response to sitting duration

Participants did not only have problems with reporting sitting in the past 7 days, but also with selecting a day. In the absence of instructions on how to select 'a day', participants choose one or another of various logically justifiable strategies, including choosing a day at random, selecting the day with the least sitting time, and choosing the day with the most sitting time. Estimates of sitting time, of course, varied, depending on the strategy chosen.

Similarly, since neither questionnaire suggests a strategy for calculating hours spent sitting during a day, participants used a variety of strategies, which also affected the estimates of sitting time. Participants found this step more challenging for IPAQ than for PASE, given its open-ended response format. This was particularly the case if their sitting time varied from day to day. Our overall impression was that it might be easier if participants were asked to report on a specific day, such as 'the total time spent sitting last Wednesday', as is used for the phone-administered IPAQ when respondents cannot answer the sitting question because their sitting times varied between days [20]. Another option would be to ask about time spent sitting 'yesterday' as respondents may find it easier to recall what they did in the last 24 hours than to recall the activities of any earlier day. These options do not overcome the problem of accurately assessing weekly sitting time if activities vary from day to day, but the suggested strategies may help respondents to focus on sitting in the last 7 days.

In general, participants found PASE's closed format easier to use, and consequently, the strategies used to formulate a response were more consistent across participants for PASE than for IPAQ. It may therefore be beneficial to use a closed format to assess sitting time in older adults, as PASE does, but to provide a greater selection of response options from which to chose, starting with 0-2 hours, and progressing in 2-hour increments, up to a response option of > 12 hours. Such an approach could decrease the time and the cognitive processing needed to complete the sitting question. Regardless of the format used, instructions should be added to give guidance in selecting a day and calculating time spent sitting, in an effort to ensure the biases in the estimates are consistently in the same direction.

### Limitations and strengths

It is usually not possible to generalize the findings of cognitive interviews to the general population because of sampling issues [12]. Although participants for this study were purposefully selected, the generalizability of the findings may be limited, because participants were generally well educated, cognitively healthy, and reported good overall health. Because education levels could influence questionnaire comprehension [14], future studies may want to target less educated people, in order to get a better understanding of how this population responds to sitting questions. As with all qualitative studies, use of the think-aloud process could have influenced the responses (i.e., reactivity effects). Answering the self-report questions in a face-to-face interview, instead of completing the questions on one's own, may also have influenced the responses. Our approach to minimizing this included not providing guidance in how to interpret and respond to the questions and probing after participants had completed the questions. Responding to questions from different questionnaires in one interview may also have affected participant's responses. The questions were therefore completed in random order. It is not likely that the order influenced the responses, as major themes were present in all transcripts.

The large sample size is a strength of this study. In general, sample sizes for studies using cognitive interviews are small, based on the assumption that problems with questionnaires will become apparent in only a small sample [12]. However, not all problems with a questionnaire may be uncovered in small samples. The findings of Blair et al. show a relationship between sample size and the number of problems with a questionnaire, with the highest number of uncovered problems for a sample size of 50 [14]. They therefore recommend larger sample sizes.

### Where to from here: continue to use self-report sitting-time measures like the IPAQ and PASE sitting-time questions?

Self-report data are more vulnerable to bias and measurement error than objective data, and quantitative studies generally report only poor to fair agreement between self-reported and objectively-measured physical activities [6] and sedentary time [28]. A potential explanation by Bauman et al. (2006) for the low validity of self report against objective measures is that questionnaires and objective measures such as accelerometers measure different aspects of physical activity behavior [6]. Bauman et al. state that sedentary time is the least well-measured type of activity [6]. Studies such as the current qualitative study that document how people respond to sitting-time questions are needed to understand what problems are occurring during the process of answering these questions, why these problems are occurring and to offer suggestions for improving these questions.

The findings of the present study may help explain the generally low to moderate agreement between self-reported and objectively-measured sitting time. One explanation could be that objective measures such as accelerometers register all sedentary activities, including brief periods that participants are unlikely to recall for self-report measures. Another explanation could be that while accelerometers register sedentary activities in all domains (e.g., leisure, work, transport), self-report measures often assess sitting time in only some domains (e.g., PASE asks about leisure-time sitting only). Finally, accelerometers register all activities with an intensity under a pre-specified cut-point, which could also include lying down and light intensity standing activities, whereas self-report questions ask about sitting specifically. Combined use of accelerometers and inclinometers could potentially overcome this problem and, if so, might be a way to obtain more valid sitting-time data.

Although some surveillance studies have started to include objective measures of sitting and PA, it is likely that researchers will continue to use self-report sitting questions. Self-report questions are convenient and inexpensive to use and therefore suitable for large population studies [6]. Furthermore, studies have shown that self-reported sitting time is associated with a range of health outcomes [3,4]. A key recommendation from a recent review on the measurement of sedentary time was for population-based studies to include both self-report measures and device-based measures [28]. Given the frequent use of sitting-time questions in these large studies, it is important to be aware of problems with self-report sitting-time questions and to improve these questions to ensure we collect the most accurate self-report sitting-time data possible. It is critical that researchers be aware that different survey instruments claiming to measure sedentary behavior may not be collecting information on the same activities and in the same domains, which should be taken into account in the interpretation of findings from different studies of sedentary behavior and health.

## Conclusion

To our knowledge, this is the first qualitative study to examine older adults' understanding of sitting-time questions from two questionnaires. The results of the present study can help researchers understand the cognitive processes that older people use to answer sitting-time questions and provide insight into the advantages and disadvantages of the two approaches for assessing sitting time. Although participants were community-dwelling adults aged 65+ years, the cognitive problems they encountered may be similar to those faced by other population groups. Respondents to sitting-time questions may be able to more accurately report on their sitting activities if our recommendations for clarifying the scope of sitting domains, providing examples relevant to older adults and suggesting strategies for formulating responses are incorporated.

This study suggests that older people face challenges when completing self-report sitting questions, which affect the accuracy of the responses. Therefore, our findings suggest that, with the types of questionnaires used in this study, we cannot be confident that we are getting accurate information about sitting time from older adults. Future research should use results from qualitative studies to inform the development and/or adjustment of questions to assess sitting time in older adults. In addition, future quantitative studies should include a criterion measure to assess validity and reliability of these questions.

## Declaration of Competing interests

The authors declare that they have no competing interests.

## Authors' contributions

JvU and WJB conceived the study. JvU and KCH developed the study design, interviewed the participants, and analyzed and interpreted the data. KCH developed the data coding and analysis process and JvU drafted the manuscript. RLH participated in conducting the interviews and analyzing and interpreting the data. All authors participated in revising manuscript drafts and in reading and approving the final manuscript.

## Pre-publication history

The pre-publication history for this paper can be accessed here:

http://www.biomedcentral.com/1471-2458/11/458/prepub
